# Supplementary material for: Heterozygous missense variant in GLI2 impairs human endocrine pancreas development
Source: Nat Commun. 2024 Mar 20;15:2483. doi: 10.1038/s41467-024-46740-8 (PMC10954617; doi:10.1038/s41467-024-46740-8)
Supplement: Supplementary file 1 — Supplementary Information [file 41467_2024_46740_MOESM1_ESM.pdf]

SUPPLEMENTARY INFORMATION

Heterozygous missense variant in *GLI2* impairs human endocrine pancreas development

Laura M. Mueller[1], Abigail Isaacson[1], Heather Wilson[1], Anna Salowka[1], Isabel Tay[1], Maolian Gong[2,3], Nancy Samir Elbarbary[4], Klemens Raile[2,3], Francesca M. Spagnoli[1]*

[1]Centre for Gene Therapy and Regenerative Medicine, King's College London, Great Maze Pond, London SE1 9RT, United Kingdom; [2]Department of Pediatric Endocrinology and Diabetology, Charité, Berlin, Germany; [3]Experimental and Clinical Research Center (ECRC), Charité Medical Faculty, Max-Delbrueck-Center for Molecular Medicine (MDC), Berlin, Germany; [4] Department of Pediatrics, Diabetes and Endocrine Unit, Faculty of Medicine, Ain Shams University, Cairo, Egypt.

*corresponding author: francesca.spagnoli@kcl.ac.uk

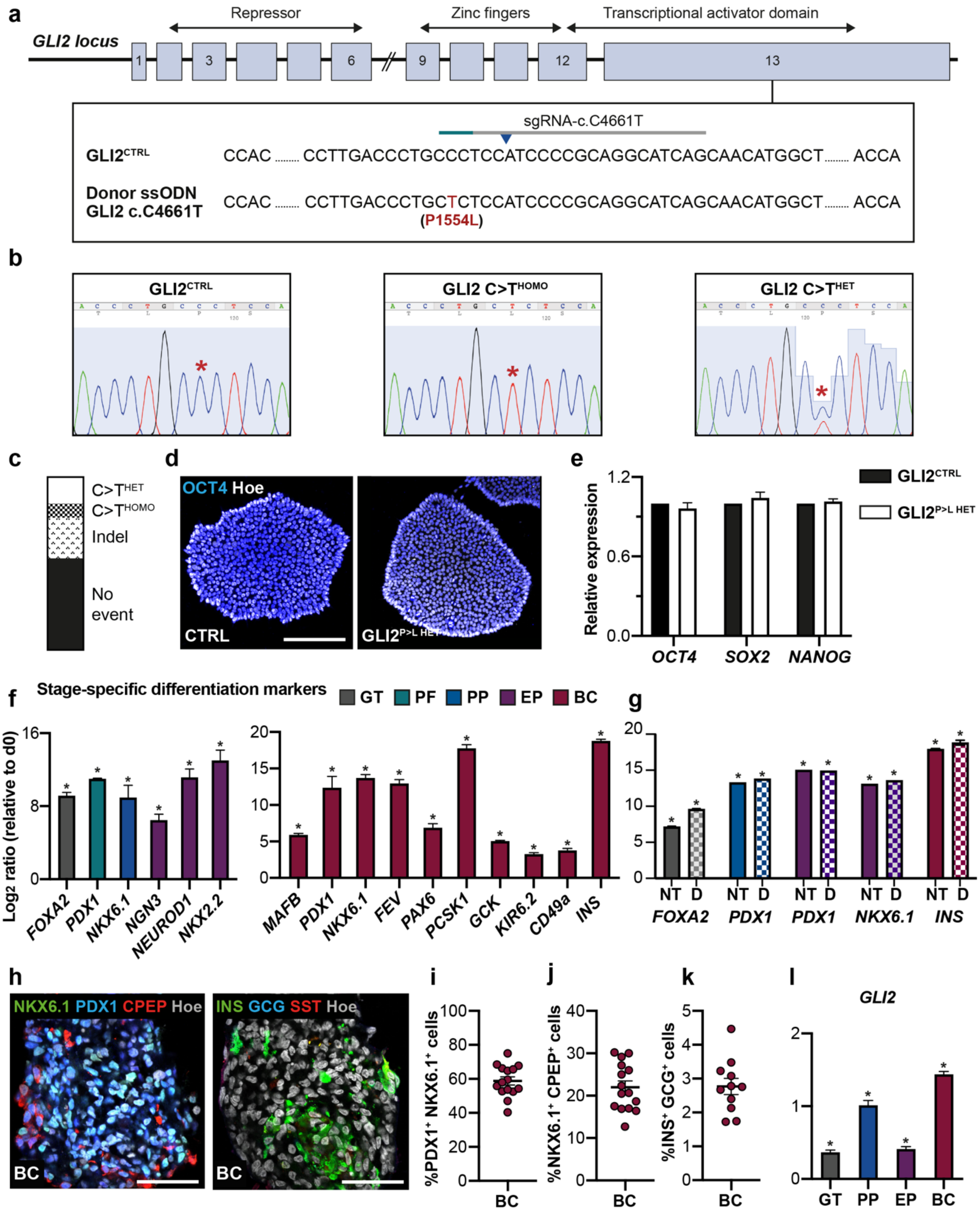

Supplementary Figure 1. Generation of patient-like GLI2 c.C4661T iPSC lines.

a CRISPR gRNA design for generating GLI2 c.C4661T disease variant using the HMGUi001-A2 iPSC line[1] (see Methods for details). The target sequence of the sgRNA and the protospacer-adjacent motif (PAM) sequences are indicated in grey and green, respectively. The mutation was introduced through homology direct repair

using a ssODN template (Supplementary Data 1). The blue arrow indicates the predicted Cas9 cleavage site.

b Sequencing-graphs of wild-type control line (GLI2^{CTRL}) and heterozygous (GLI2$^{P>L\ HET}$) and homozygous (GLI2$^{P>L\ HOMO}$) c.C4661T mutant clones. Red asterisk indicates C>T switch.

c CRISPR Cas9 efficiency. We established several heterozygous (14.71% efficiency) and homozygous (8.82% efficiency) c.C4661T mutant iPSC clones, as confirmed by Sanger sequencing.

d Representative immunofluorescence (IF) staining images for OCT4 on GLI2^{CTRL} and GLI2$^{P>L\ HET}$ iPSC clones. IF experiments were performed 2 independent times, and each experiment showed similar results. Nuclei were labeled with Hoechst (Hoe).

e Representative RT-qPCR of selected pluripotency gene transcripts in GLI2^{CTRL} and CRISPR-Cas9 engineered GLI2$^{P>L\ HET}$ iPSC lines. Three independent RT-qPCR experiments (n=3) were performed on GLI2^{CTRL} and two GLI2$^{P>L\ HET}$ independent clones, and each experiment showed similar results. *E*xpression values were normalized to GAPDH and shown as relative levels to GLI2^{CTRL} set to '1'. Values are shown are mean ± SEM.

f Representative RT-qPCR of stage-specific markers in iPSC-based pancreatic differentiation model. Values are normalized to *GAPDH* and shown as Log2 ratio relative to day 0. Values shown are mean ± SEM. Five independent RT-qPCR experiments (n=5) were performed on GLI2^{CTRL} cells, and each experiment showed similar results. Statistical significance calculated using two-tailed Student's t-test. *$P<0.05$. GT, gut tube endoderm; PF, pancreatic foregut; PP, pancreatic progenitors; EP, endocrine progenitors; BC, β-like cells.

g Representative RT-qPCR analysis of stage-specific markers in GLI2^{CTRL} iPSCs differentiated after doxycycline (D) treatment or non-treated (NT). Values were normalized to *GAPDH* and shown as Log2 ratio relative to day 0. Two independent RT-qPCR experiments (n=2) were performed on (D) treated or non-treated (NT) GLI2^{CTRL} cells, and each experiment showed similar results. Statistical significance calculated using two-tailed Student's t-test. *P<0.05.

h Representative immunofluorescence staining images of differentiated β-like cell (BC) clusters at day 21 for PDX1, NKX6.1, human C-PEPTIDE (CPEP) and INSULIN (INS), GLUCAGON (GCG), SOMATOSTATIN (SST). Nuclei were labeled with Hoechst (Hoe). Scale bar, 20 μm.

i Scatter plot shows percentage (%) of cells double positive for PDX1 and NKX6.1 in differentiated BC clusters.

j Scatter plot shows percentage (%) of cells double positive for NKX6.1 and human C-PEPTIDE at day 21.

k Scatter plot shows percentage (%) INSULIN/GLUCAGON-double positive cells, representing polyhormonal cell fraction, at day 21. The number of positive cells in (**i, j, k**) staining was normalized to the total number of cells contained in each cluster and shown as %. Values shown are mean ± SEM.

l Representative RT-qPCR of *GLI2* expression in iPSC-based differentiation model. Values were normalized to *GAPDH* and shown relative to day 0. Statistical significance calculated using two-tailed Student's t-test. *P<0.05. Source data are provided as a Source Data file.

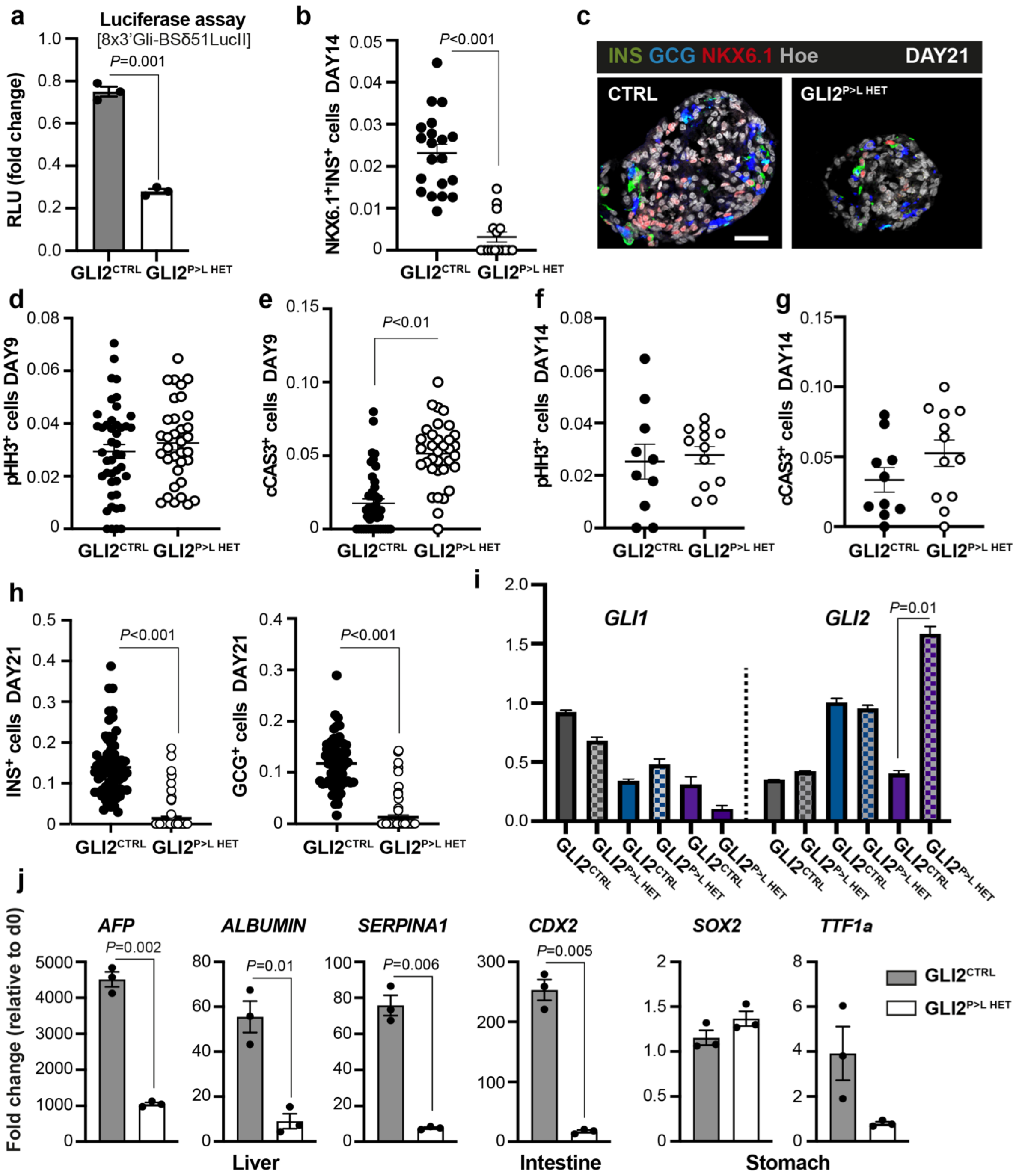

Supplementary Figure 2. Characterization of GLI2$^{P>L\ HET}$-derived iPSCs during pancreatic cell differentiation.

a GLI luciferase assay in GLI2^{CTRL} and GLI2$^{P>L\ HET}$ iPSCs. Results were normalized for transfection efficiency using Renilla luciferase and are represented as Firefly/Renilla activity ratio. *RLU* refers to relative luminescence units. The experiment was performed 3 times. Values shown are mean ± SD. Statistical significance calculated using two-tailed Student's t-test; *P*=0.001.

b Scatter plot shows significant decrease of NKX6.1- and INSULIN-double positive cells in differentiated GLI2$^{P>L\ HET}$ clusters. The number of NKX6.1/INSULIN^{+} cells was normalized to the total number of cells contained in each cluster and shown as %. n=3 differentiation experiments on two GLI2$^{P>L\ HET}$ independent clones. Two-tailed Student's t-test; P<0.001.

c Representative immunofluorescence (IF) staining images for indicated markers on GLI2^{CTRL}- and GLI2$^{P>L\ HET}$-derived clusters at day 21. IF experiments were performed 3 independent times, and each experiment showed similar results. Nuclei were labeled with Hoechst (Hoe). Scale bar, 30 µm.

d-g Quantification of the cells positive for the mitotic marker phosho-histone H3 (pHH3) and active cleaved caspase-3 (cCAS3) at day 9 (d, e) and day 14 (f, g) in differentiated GLI2^{CTRL} and GLI2$^{P>L\ HET}$ clusters. n=3. Two-tailed Student's t-test; P<0.01 in **(e)**.

h Scatter plot shows significant decrease of INSULIN (INS)$^{+}$ and GLUCAGON (GCG)$^{+}$ cells in differentiated GLI2^{CTRL} and GLI2$^{P>L\ HET}$ clusters at day 21. The number INS^{+} and GCG^{+} was normalized to the total number of cells contained in each cluster and shown as %. n = 3. Two-tailed Student's t-test; P<0.001.

i RT-qPCR analysis of *GLI1* and *GLI2* during differentiation into endoderm (grey), pancreatic (blue) and endocrine progenitors (purple) from GLI2^{CTRL} and GLI2$^{P>L\ HET}$ iPSCs. Values were normalized to *GAPDH* and shown as fold change relative to undifferentiated cells (d0). Values shown are mean ± SEM. n = 3. Two-tailed Student's t-test; P< 0.01.

j RT-qPCR analysis of selected markers of endodermal-derivative lineages in GLI2^{CTRL} and GLI2$^{P>L\ HET}$ differentiated cells at day (d) 9. Values were normalized to *GAPDH* and shown as fold change relative to undifferentiated cells (d0). Values shown are mean ± SEM. n=3. Two-tailed Student's t-test; P values are reported in the figure. Source data are provided as a Source Data file.

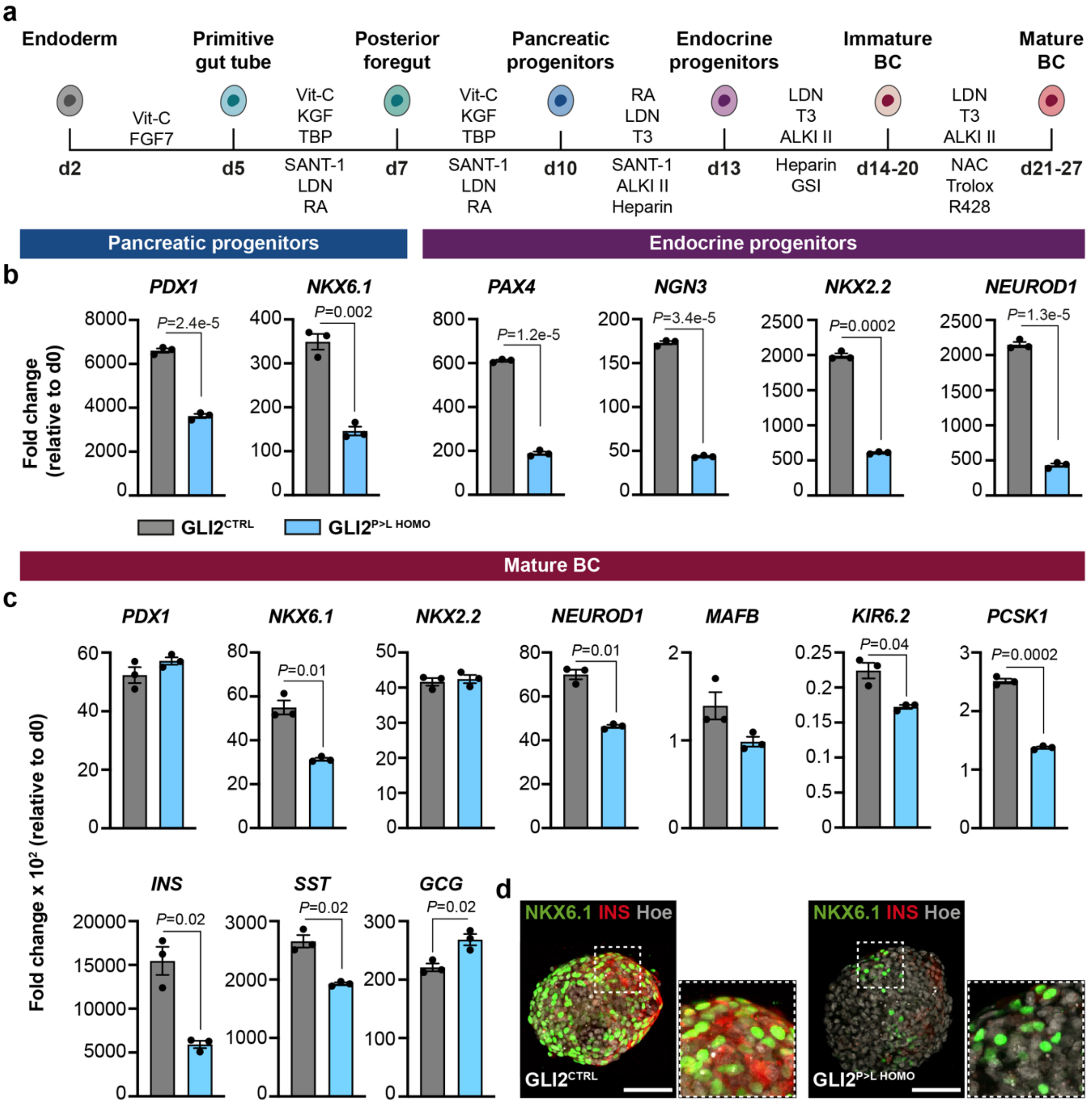

Supplementary Figure 3. Directed differentiation of GLI2$^{P>L\ HOMO}$ iPSCs into β-like cells using an independent differentiation protocol.

a Schematic diagram of the protocol[3] used to differentiate GLI2^{CTRL} and GLI2$^{P>L\ HOMO}$ iPSCs into β-like cells (BC). For consistency with the previously employed protocol[2], we adapted the seven-stage protocol from Rezania et al.[3] to a 3D in suspension one and cells were differentiated on an orbital shaker. This protocol requires the addition of the HH inhibitor SANT-1 to the differentiation medium for eight consecutive days from posterior foregut stage onwards until the endocrine progenitor stage[3].

b, c Representative RT-qPCR analyses of indicated stage-specific markers. Values were normalized to GAPDH and shown as fold change relative to undifferentiated cells (d0). Values shown are mean ± SEM. n=2 differentiation experiments on two GLI2$^{P>L}$

^{HOMO} independent clones. Two-tailed Student's t-test; exact *P* values are reported in the figure. Source data are provided as a Source Data file.

d Representative whole-mount immunostaining for NKX6.1 and INSULIN in $GLI2^{CTRL}$ and $GLI2^{P>L\ HOMO}$ iPSC-derived BC clusters at day 27. Nuclei were labeled with Hoechst (Hoe). Scale bars, 25 μm.

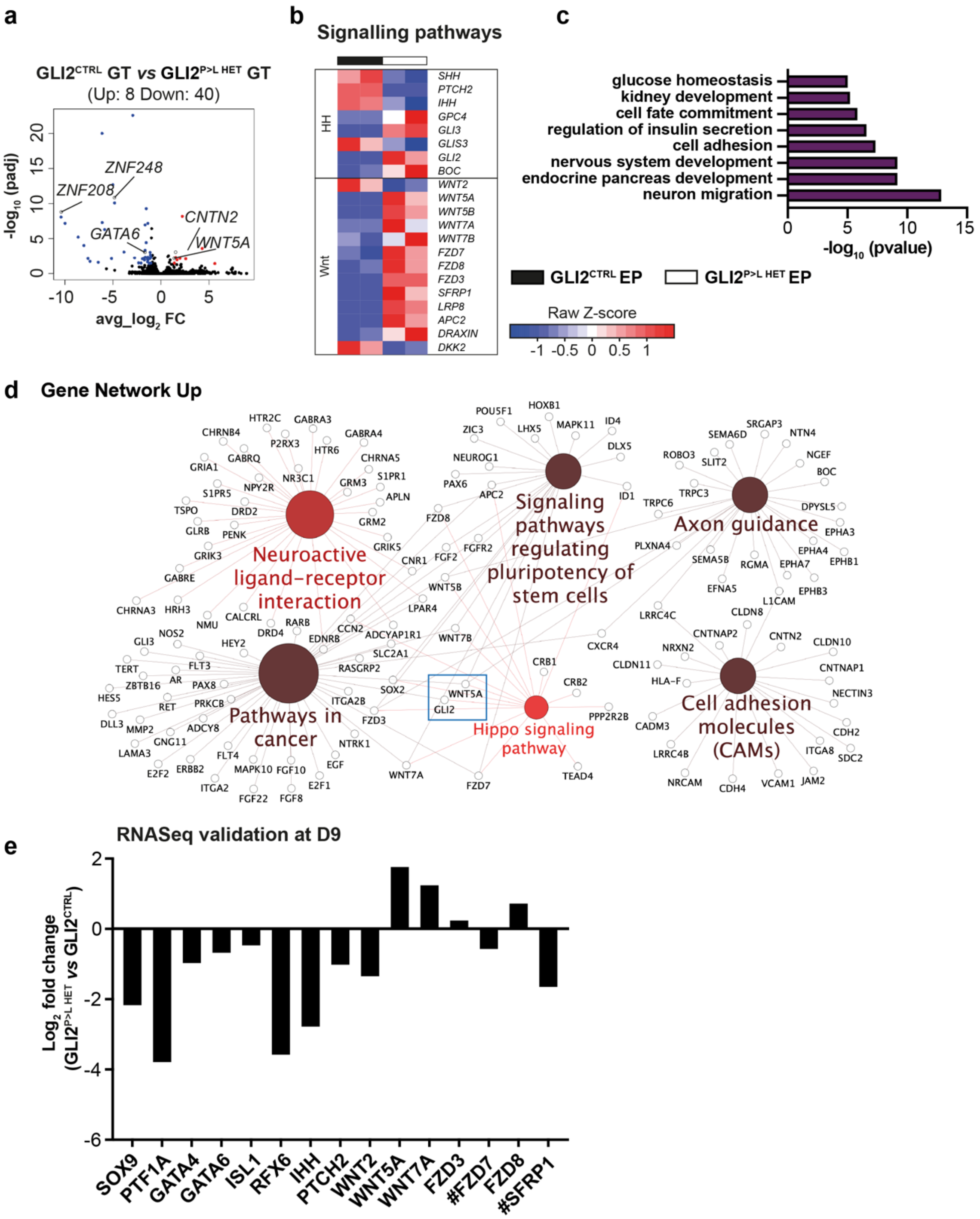

Supplementary Figure 4. RNA-Seq analysis of GLI2^{CTRL} and GLI2$^{P>L\ HET}$ iPSCs undergoing differentiation into endocrine progenitor cells.

a Volcano plots visualizing the global transcriptional change across the groups compared [Gut Tube (GT) D5 GLI2^{CTRL} *vs* GLI2$^{P>L\ HET}$]. Each data point in the plot represents a gene. Genes with an adjusted *P* value less than 0.05 and a log2 fold change greater than 1 are indicated by red dots. These represent up-regulated genes. Genes with an adjusted *P* value less than 0.05 and a log2 fold change less than -1 are indicated by blue dots. These represent down-regulated genes. Genes shown are

among the 23 ones similarly dysregulated in GLI2$^{P>L\ HET}$ at both GT and Endocrine Progenitor (EP) stages. Wald test was used as statistical test as implemented in DESeq2 package (see Methods).

b Heatmap of selected differentially expressed genes between GLI2$^{P>L\ HET}$- and GLI2^{CTRL}-derived EPs. Boxes highlight genes belonging to the indicated signaling pathways. Colours represent high (red) or low (blue) expression values based on Z-score normalized to FPKM values for each gene.

c Gene ontology (GO) enrichment analysis of differentially expressed genes ($p<0.05$) performed on EP RNA-Seq datasets. GO Biological Process (BP) categories are shown.

d GO term enrichment and pathway term network analysis of DEGs between GLI2^{CTRL}- and GLI2$^{P>L\ HET}$-derived EPs. Gene Network showing up-regulated genes (FDR ≦ 0.05). Term node size increases with term significance and colour corresponds to a particular functional group that is based on the similarity of their associated genes. Groups that shared 50% or more of the same genes were merged. The proportion of each node that is filled with colour reflects the kappa score. Functionally related groups partially overlap.

e RT-qPCR validation of a subset of DEGs in GLI2^{CTRL}- and GLI2$^{P>L\ HET}$-derived pancreatic progenitors at day (D) 9. # indicate genes with discordant regulation at EP stage. Data were normalized to that of GAPDH and shown as Log2-expression ratio between GLI2$^{P>L\ HET}$ and GLI2^{CTRL} EP cells. Source data are provided as a Source Data file.

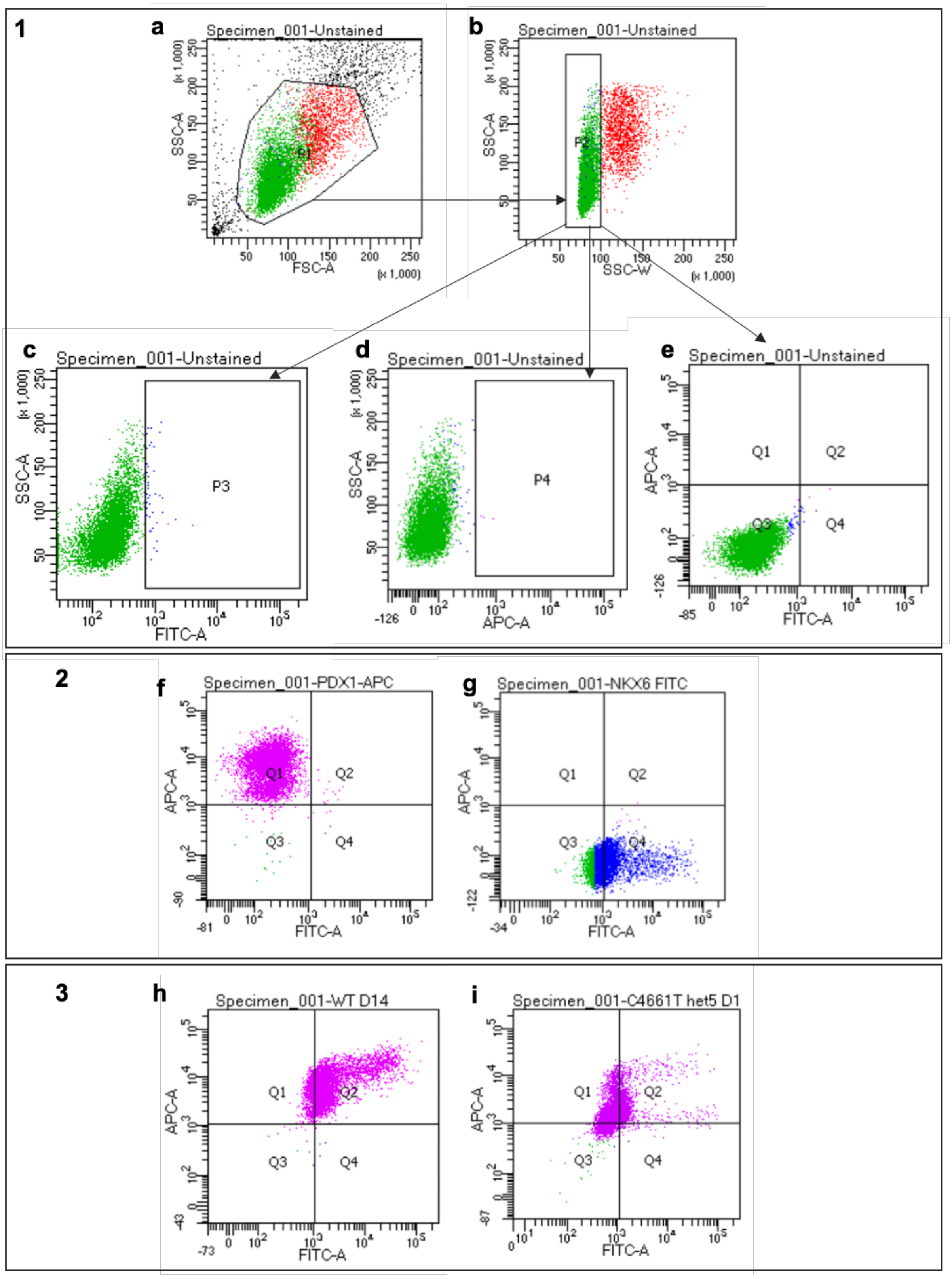

Supplementary Figure 5. Representative flow cytometry plots and gating strategy.

Representative flow cytometry pseudocolor plots and gating strategy for PDX1⁺/NKX6.1⁺ populations related to Figure 2 and Figure 3.

BOX 1. Outline of gating strategy for unstained cells as a negative control. A population of cells (P1) was selected based on size (FSC-A) and granularity (SSC-A) to exclude debris (**a**). Doublets and multiplets (red) were excluded from population P1 based on side scatter area (SSC-A) and side scatter width (SSC-W) giving rise to population P2 consisting of single cells (**b**). The population of single cells (P2) was then plotted against FITC-A (**c**) and APC-A (**d**) to select the populations positive for FITC-A (P3) and APC-A (P4). Lastly, a two-parameter density plot of APC-A *versus* FITC-A, allowing visualization of single cells positive for both markers, was plotted. Based on this plot a gate was set to isolate unstained cells negative for APC-A and FITC-A (**e**).

BOX 2. Final plots depicting single cells stained for PDX1 only and NKX6.1 only. Equivalent gating strategy was performed to derive populations of single cells positive for PDX1 (**f**) and NKX6.1 (**g**). Gate from **e** was applied to **f** and **g** to ensure that GLI2^{CTRL} stained cells were gated against unstained cells.

BOX 3. GLI2^{CTRL} (**h**) and GLI2$^{P>L\ HET}$ (**i**) cells stained for PDX1 and NKX6.1 were gated (as shown in a-e) to obtain a population of single cells composed of PDX1^{+} (Q1), NKX1^{+}(Q4), PDX1^{+}NKX6.1^{+}(Q2) and unstained cells (Q3). The gate separating the four populations is the same as in **e**, **f** and **g**.

Supplementary Table 1. *In silico* pathogenicity predictions of the heterozygous *GLI2* p.P1554L variant^.

Tool	Prediction
**SIFT**[1]	Deleterious
**PolyPhen2**[2]	Probably Damaging
**PROVEAN**[3]	Deleterious
**CONDEL**[4]	Deleterious
**CADD PHRED**[5]	28.4

^The missense variant **rs767802807** is annotated as of uncertain significance and there is no functional evidence for this variation in ClinVar [VCV001506585.4]. Several heterozygous *GLI2* mutations have been reported to cause forebrain and pituitary defects.[4-6] (see https://omim.org/allelicVariants/165230)
However, these symptoms were not present in any of the sequenced patients.
[1]https://sift.bii.a-star.edu.sg/www/SIFT_seq_submit2.html;
[2]http://genetics.bwh.harvard.edu/pph2/;
[3]http://provean.jcvi.org/index.php;
[4]https://bbglab.irbbarcelona.org/fannsdb/help/condel.html;
[5]https://cadd.gs.washington.edu/.

SUPPLEMENTARY REFERENCES

1 Yumlu S, Stumm J, Bashir S, Dreyer A-K, Lisowski P, Danner E, et al. Gene editing and clonal isolation of human induced pluripotent stem cells using CRISPR/Cas9. *Methods* **121–122**, 29–44 (2017).

2 Russ HA, Parent AV, Ringler JJ, Hennings TG, Nair GG, Shveygert M, et al. Controlled induction of human pancreatic progenitors produces functional beta-like cells in vitro. *EMBO J* **34**, 1759–1772 (2015).

3 Rezania A *et al.* Reversal of diabetes with insulin-producing cells derived in vitro from human pluripotent stem cells. *Nat Biotechnol.* **32**, 1121–1133 (2014).

4 Bertolacini CDP, Ribeiro-Bicudo LA, Petrin A da S, Richieri-Costa A & JC., M. Clinical findings in patients with GLI2 mutations – phenotypic variability. *Clin Genet.* **81**, 70–75 (2012).

5 Flemming GMC, Klammt J, Ambler G, Bao Y, Blum WF, Cowell C, et al. Functional Characterization of a Heterozygous GLI2 Missense Mutation in Patients With Multiple Pituitary Hormone Deficiency. *J Clin Endocrinol Metab.* **98**, E567–575 (2013).

6 Roessler, E. *et al.* Loss-of-function mutations in the human GLI2 gene are associated with pituitary anomalies and holoprosencephaly-like features. *Proceedings of the National Academy of Sciences* **100**, 13424–13429 (2003).
