# Supplementary material for: Heterozygous missense variant in GLI2 impairs human endocrine pancreas development
Source: Nat Commun. 2024 Mar 20;15:2483. doi: 10.1038/s41467-024-46740-8 (PMC10954617; doi:10.1038/s41467-024-46740-8)
Supplement: Supplementary file 3 — Description of Additional Supplementary Files [file 41467_2024_46740_MOESM3_ESM.pdf]

Description of Additional Supplementary Files

File Name: Supplementary Data 1
Description: Tables of primers.

File Name: Supplementary Data 2
Description: Tables of antibodies and cytokines.

File Name: Supplementary Data 3
Description: DEG RNASeq D0 iPSCs CTRL vs MUT.

File Name: Supplementary Data 4
Description: DEG RNASeq D5 iPSCs.

File Name: Supplementary Data 5
Description: DEG RNASeq D14 iPSCs CTRL vs MUT.
