# Supplementary material for: Heterozygous missense variant in GLI2 impairs human endocrine pancreas development
Source: Nat Commun. 2024 Mar 20;15:2483. doi: 10.1038/s41467-024-46740-8 (PMC10954617; doi:10.1038/s41467-024-46740-8)
Supplement: Supplementary file 9 — Reporting Summary [file 41467_2024_46740_MOESM9_ESM.pdf]

nature portfolio

Corresponding author(s): Spagnoli, FM

Last updated by author(s): Feb 27, 2024

Reporting Summary

Nature Portfolio wishes to improve the reproducibility of the work that we publish. This form provides structure for consistency and transparency in reporting. For further information on Nature Portfolio policies, see our Editorial Policies and the Editorial Policy Checklist.

Statistics

For all statistical analyses, confirm that the following items are present in the figure legend, table legend, main text, or Methods section.

n/a	Confirmed	
☐	☒	The exact sample size (*n*) for each experimental group/condition, given as a discrete number and unit of measurement
☐	☒	A statement on whether measurements were taken from distinct samples or whether the same sample was measured repeatedly
☐	☒	The statistical test(s) used AND whether they are one- or two-sided<br>*Only common tests should be described solely by name; describe more complex techniques in the Methods section.*
☒	☐	A description of all covariates tested
☐	☒	A description of any assumptions or corrections, such as tests of normality and adjustment for multiple comparisons
☐	☒	A full description of the statistical parameters including central tendency (e.g. means) or other basic estimates (e.g. regression coefficient) AND variation (e.g. standard deviation) or associated estimates of uncertainty (e.g. confidence intervals)
☐	☒	For null hypothesis testing, the test statistic (e.g. *F*, *t*, *r*) with confidence intervals, effect sizes, degrees of freedom and *P* value noted<br>*Give P values as exact values whenever suitable.*
☒	☐	For Bayesian analysis, information on the choice of priors and Markov chain Monte Carlo settings
☒	☐	For hierarchical and complex designs, identification of the appropriate level for tests and full reporting of outcomes
☒	☐	Estimates of effect sizes (e.g. Cohen's *d*, Pearson's *r*), indicating how they were calculated

Our web collection on statistics for biologists contains articles on many of the points above.

Software and code

Policy information about availability of computer code

Data collection	Confocal microscopy: Zeiss LSM 710, Zen 3.9 version software<br>qPCR: LightCycler 96 system (Roche)
Data analysis	Data and Statistical Analysis: Excel (Microsoft 365)<br>Data and Statistical Analysis: Prism (Graphpad version 8)<br>Image Analysis: Image J (2.2)/Fiji (NIH)<br>Image Analysis: Imaris (Bitplane version 8)<br>Flow cytometry data: FlowJo v10 software<br>RNASeq: STAR (v.2.5.2.b), R suite (v3.6.0, http://www.R-project.org/), Cufflinks 2.2.1, DAVIS, Cytoscape (v3.8.0), GSEA software package (Desktop v4.1.0)

For manuscripts utilizing custom algorithms or software that are central to the research but not yet described in published literature, software must be made available to editors and reviewers. We strongly encourage code deposition in a community repository (e.g. GitHub). See the Nature Portfolio guidelines for submitting code & software for further information.

Data

Policy information about availability of data

All manuscripts must include a data availability statement. This statement should provide the following information, where applicable:

- Accession codes, unique identifiers, or web links for publicly available datasets
- A description of any restrictions on data availability
- For clinical datasets or third party data, please ensure that the statement adheres to our policy

The RNA-sequencing data of the human iPSC lines (Control and GLI2P>LHET mutant) have been deposited in GEO [accession number GSE224943] and is publicly available. Trimmed reads were mapped to the Homo sapiens GRCh38p13 reference genome No original code was reported in this study. Source data are provided with this paper.

Human research participants

Policy information about studies involving human research participants and Sex and Gender in Research.

Reporting on sex and gender	For this study human fetal pancreatic tissue was used that was obtained from the MRC/Wellcome Trust-funded Human Developmental Biology Resource (HDBR; https://www.hdbr.org) following overnight fixation in 4% paraformaldehyde (PFA). The tissue samples were then processed for cryosectioning, immunostaining and imaging at King's College London. The tissue was deidentified and population characteristics were not provided. Information on sex and gender about the family is included in Methods and Supplementary Table 1.
Population characteristics	N/A
Recruitment	We did not recruit human subjects for this study.
Ethics oversight	All work was undertaken in approval of the HDBR Steering Committee to the Spagnoli lab. at King's College London, UK (License #200523) and Charité committee on human research approved the study (EA-No EA2/054/11).

Note that full information on the approval of the study protocol must also be provided in the manuscript.

Field-specific reporting

Please select the one below that is the best fit for your research. If you are not sure, read the appropriate sections before making your selection.

☒ Life sciences ☐ Behavioural & social sciences ☐ Ecological, evolutionary & environmental sciences

For a reference copy of the document with all sections, see nature.com/documents/nr-reporting-summary-flat.pdf

Life sciences study design

All studies must disclose on these points even when the disclosure is negative.

Sample size	No statistical methods were used to predetermine sample sizes. The sample size was chosen based on previous experience in the laboratory and the literature (n= 3-6). All n values are clearly stated in Figure legends.
Data exclusions	No data were excluded from the analysis.
Replication	All experiments were repeated at least three independent times with independent samples to verify the reproducibility of the experimental findings (precisely stated in the figure legends). The results obtained were always similar.
Randomization	The experiments were not randomized.
Blinding	The investigators were not blinded to allocation during experiments and outcome assessment. Computational analysis of the RNASeq datasets and image analyses were blinded. Blinding was not performed because values derived from all experiments were quantitative and did not require subjective interpretation.

Reporting for specific materials, systems and methods

We require information from authors about some types of materials, experimental systems and methods used in many studies. Here, indicate whether each material, system or method listed is relevant to your study. If you are not sure if a list item applies to your research, read the appropriate section before selecting a response.

Materials & experimental systems

n/a	Involved in the study	
	☒	Antibodies
	☒	Eukaryotic cell lines
☒		Palaeontology and archaeology
☒		Animals and other organisms
☒		Clinical data
☒		Dual use research of concern

Methods

n/a	Involved in the study	
☒		ChIP-seq
	☒	Flow cytometry
☒		MRI-based neuroimaging

Antibodies

Antibodies used: See Supplementary Data 2 in Supplementary Information.

Validation: Validation:
anti-cCas3: DOI: 10.1038/s41467-018-07474-6; https://www.cellsignal.co.uk/products/primary-antibodies/cleaved-caspase-3-asp175-antibody/9661
anti-C-peptide: https://www.cellsignal.com/products/primary-antibodies/c-peptide-antibody/4593
anti-E-cadherin: DOI: 10.1038/s41467-018-07474-6; https://www.sigmaaldrich.com/GB/en/product/sigma/u3254 10.1038/s41467-018-07474-6
anti-Gli2 (Abcam): DOI: 10.1016/j.devcel.2015.09.001 ; https://www.abcam.com/products/primary-antibodies/gli2-antibody-ab7195.html
anti-Pdx1: DOI: 10.1038/s41467-018-07474-6; https://www.abcam.com/pdx1-antibody-ab47308.html
anti-pH3 (Millipore): DOI: 10.1038/s41467-018-07474-6; https://www.merckmillipore.com/GB/en/product/Anti-phospho-Histone-H3-Ser10-Antibody-Mitosis-Marker,MM_NF-06-570
anti-Somatostatin (Santa Cruz): https://www.scbt.com/p/somatostatin-antibody-d-20
anti-Nkx6.1: https://dshb.biology.uiowa.edu/F55A10
anti-Glucagon (Immunostar Inc.): https://www.immunostar.com/product/glucagon-antibody/
anti-Insulin (Thermofisher): https://www.thermofisher.com/antibody/product/Insulin-Antibody-Polyclonal/PA1-26938

Eukaryotic cell lines

Policy information about cell lines and Sex and Gender in Research

Cell line source(s): HMGUi001-A2 (sex: female; gift of Kuhn lab, MDC, Berlin, DE); HEK293T (ATTC #CRL-3216)

Authentication: RRID:CVCL_ZA40; RRID:CVCL_0063

Mycoplasma contamination: All cell lines tested negative for mycoplasma contamination which was carried out routinely.

Commonly misidentified lines (See ICLAC register): No commonly misidentified cell lines were used in the study

Flow Cytometry

Plots

Confirm that:

☒ The axis labels state the marker and fluorochrome used (e.g. CD4-FITC).

☒ The axis scales are clearly visible. Include numbers along axes only for bottom left plot of group (a 'group' is an analysis of identical markers).

☒ All plots are contour plots with outliers or pseudocolor plots.

☒ A numerical value for number of cells or percentage (with statistics) is provided.

Methodology

Sample preparation: For flow-based analysis, dissociated cell clusters were fixed for 20 min at 4°C in cold BD fixation/permeabilizationTM solution (BD Bioscience). Cells were washed twice in BD Perm/WashTM Buffer (BD Bioscience) and incubated with primary antibodies in the dark for 2 hrs at 4°C. Cells were washed 3x in BD WashTM Buffer (BD Bioscience), resuspended in BD-FBS stainingTM buffer (BD Bioscience) with secondary antibodies and incubated for 1 hr at RT.

Instrument: FACS Aria II.

Software: FlowJo software was used to analyze data.

Cell population abundance	Data for 10,000 events in the post-sorted fraction were recorded for each sample. Purity was determined as falling within gated regions determined to exclude negative control (isotype-stained) samples.
Gating strategy	Negative (isotype-stained) controls were used for all flow cytometry samples.

☒ Tick this box to confirm that a figure exemplifying the gating strategy is provided in the Supplementary Information.
